# Indications for Inpatient Magnetoencephalography in Children – An Institution’s Experience

**DOI:** 10.3389/fnhum.2021.667777

**Published:** 2021-06-04

**Authors:** Michael W. Watkins, Ekta G. Shah, Michael E. Funke, Stephanie Garcia-Tarodo, Manish N. Shah, Nitin Tandon, Fernando Maestu, Christopher Laohathai, David I. Sandberg, Jeremy Lankford, Stephen Thompson, John Mosher, Gretchen Von Allmen

**Affiliations:** ^1^Division of Child Neurology, Department of Pediatrics, McGovern Medical School, Houston, TX, United States; ^2^Department of Neurology, McGovern Medical School, Houston, TX, United States; ^3^Pediatric Neurology Unit, Children’s Hospital, Geneva University Hospitals, Geneva, Switzerland; ^4^Department of Neurosurgery, McGovern Medical School, Houston, TX, United States; ^5^Division of Pediatric Neurosurgery, Department of Pediatric Surgery, McGovern Medical School, Houston, TX, United States; ^6^Laboratory of Cognitive and Computational Neuroscience, Center for Biomedical Technology, Universidad Complutense and Universidad Politecnica de Madrid, Madrid, Spain; ^7^Department of Experimental Psychology, Universidad Complutense de Madrid, Madrid, Spain

**Keywords:** magnetoencephalography, presurgical epilepsy evaluation, inpatient MEG, intractable epilepsy, SRSE, epilepsy surgery, pediatric epilepsy

## Abstract

Magnetoencephalography (MEG) is recognized as a valuable non-invasive clinical method for localization of the epileptogenic zone and critical functional areas, as part of a pre-surgical evaluation for patients with pharmaco-resistant epilepsy. MEG is also useful in localizing functional areas as part of pre-surgical planning for tumor resection. MEG is usually performed in an outpatient setting, as one part of an evaluation that can include a variety of other testing modalities including 3-Tesla MRI and inpatient video-electroencephalography monitoring. In some clinical circumstances, however, completion of the MEG as an inpatient can provide crucial ictal or interictal localization data during an ongoing inpatient evaluation, in order to expedite medical or surgical planning. Despite well-established clinical indications for performing MEG in general, there are no current reports that discuss indications or considerations for completion of MEG on an inpatient basis. We conducted a retrospective institutional review of all pediatric MEGs performed between January 2012 and December 2020, and identified 34 cases where MEG was completed as an inpatient. We then reviewed all relevant medical records to determine clinical history, all associated diagnostic procedures, and subsequent treatment plans including epilepsy surgery and post-surgical outcomes. In doing so, we were able to identify five indications for completing the MEG on an inpatient basis: (1) super-refractory status epilepticus (SRSE), (2) intractable epilepsy with frequent electroclinical seizures, and/or frequent or repeated episodes of status epilepticus, (3) intractable epilepsy with infrequent epileptiform discharges on EEG or outpatient MEG, or other special circumstances necessitating inpatient monitoring for successful and safe MEG data acquisition, (4) MEG mapping of eloquent cortex or interictal spike localization in the setting of tumor resection or other urgent neurosurgical intervention, and (5) international or long-distance patients, where outpatient MEG is not possible or practical. MEG contributed to surgical decision-making in the majority of our cases (32 of 34). Our clinical experience suggests that MEG should be considered on an inpatient basis in certain clinical circumstances, where MEG data can provide essential information regarding the localization of epileptogenic activity or eloquent cortex, and be used to develop a treatment plan for surgical management of children with complicated or intractable epilepsy.

## Introduction

Magnetoencephalography (MEG) is a direct, non-invasive, neurophysiologic study that provides complimentary interictal and ictal data to electroencephalography (EEG) by recording and localizing the magnetic fields generated by brain activity in real time. MEG is particularly sensitive to epileptogenic sources that arise from the cerebral sulci and large cortical planes as they are of tangential orientation (Bagić and Bowyer, 2017). The importance of source orientation is emphasized by the fact that nearly 35% of EEG negative cases can exhibit exclusive discharges on MEG (Shibasaki et al., 2007). As opposed to EEG, which measures electrical activity of the brain, MEG detects the associated magnetic fields, which are not distorted by surrounding tissues. For this reason, MEG is particularly useful in patients with altered brain anatomy or a history of neurosurgical intervention. A number of evidence-based indications for the use of MEG in the evaluation of patients with epilepsy have recently been outlined (Bagić et al., 2020). Overall, when used in combination with scalp EEG and magnetic resonance imaging (MRI), MEG can be an integral component of the pre-surgical epilepsy evaluation, providing additional information in 35% of cases (Stefan et al., 2003; Sutherling et al., 2008) and preventing a significant number of patients from being categorized as non-surgical.

Magnetoencephalography is recognized as a valuable non-invasive clinical method for localization of the epileptogenic zone and critical functional areas in as part of a pre-surgical evaluation in patients with intractable epilepsy (Kharkar and Knowlton, 2015). MEG is also useful in localizing functional areas as part of pre-surgical planning for tumor resection (Bagić et al., 2017). Due to extensive spatial sampling with modern systems now including more than 300 specialized sensors distributed over the surface of the head, source localization accuracy with MEG can be high, reaching 2 to 3 mm (Bagić and Bowyer, 2017), but this relationship is variably dependent on the size and pathology of the underlying epileptic lesion (Bast et al., 2004; Widjaja et al., 2008; Mu et al., 2014). A high level of data concordance between MEG and these studies, as well as with invasive investigatory techniques such as stereoelectroencephalography (SEEG) or electrocorticography (ECoG), is associated with more favorable surgical outcomes (Cuello-Oderiz et al., 2015; Murakami et al., 2016). MEG can be very helpful in localization of non-lesional, multi-lesional, and extratemporal epilepsies, which tend to be especially prevalent in children, as approximately 50% of surgical cases in children have an underlying etiology of dysplasia or brain malformation (Jung et al., 2013; Albert et al., 2014). Because of the difficulty in localization of the seizure onset zone that is often a hallmark of these cases in children, more data points from different methodologies are often required for rigorous surgical decision-making. For pediatric epilepsy groups such as ours, MEG is an essential component in the majority of our pre-surgical evaluations, and can be especially useful if the other components of the pre-surgical evaluation have provided discordant or inconclusive localization hypotheses, where MEG is then needed as a “tie-breaker” (Alkawadri et al., 2013). In these cases, MEG can contribute to the decision for whether to proceed to surgery, type of surgery or surgical approach, including the need for invasive EEG monitoring (Garcia-Tarodo et al., 2018; Bagić et al., 2020).

In most cases MEG is performed on an outpatient basis, as part of a pre-surgical evaluation that can include a variety of other testing modalities such as 3T MRI, positron emission tomography (PET), inpatient video-EEG monitoring, and single photon emission computed tomography (SPECT). This is reasonable practice given that most pre-surgical evaluations are in patients with epilepsy that is chronic but fairly stable, although intractable, and it is safe and convenient to plan the tests needed as an outpatient over a series of weeks or even months. The other reason MEG is routinely performed as an outpatient in the aforementioned group of patients is that, in the fee-for-service healthcare environment that currently exists in the U.S., insurance reimbursement for MEG that is not based on DRG (diagnosis related groups) is necessary to assure economic sustainability for most MEG centers (American Academy of Neurology, 2009). However, there are clearly instances, although in the minority of cases, where MEG is indicated as an inpatient test. The most obvious instances are those in which the epilepsy becomes acutely or sub-acutely life-threatening, such as ongoing or repeated episodes of status epilepticus. But there are also somewhat less emergent instances where risks outweigh benefits, to both the patient and the MEG center, of waiting for elective outpatient testing to occur, or where there is no economic advantage to performing the test as an outpatient. One study has reported use of MEG for surgical treatment of refractory status epilepticus in 5 pediatric patients (Mohamed et al., 2007), but there are no published reports in the literature that further explore the broader question of when MEG is indicated as an inpatient test. Here, we report our recent institutional experience of inpatient MEG in children, and discuss 5 indications for inpatient MEG evaluation.

## Materials and Methods

All MEG studies performed at our MEG Center at Children’s Memorial Hermann Hospital in Houston, TX, United States between January 2012 and December 2020 were retrospectively reviewed. A total 34 children who had undergone inpatient MEG were identified. Relevant medical data, including hospitalizations and hospital course, epilepsy history, seizure semiology, scalp EEG data, neuroimaging studies, surgical procedures, and outpatient clinic notes were reviewed as appropriate, and for those who had subsequent neurosurgical procedures performed at our institution, post-surgical outcome and pathology were also reviewed. The seizure semiologies at the time of inpatient MEG were categorized according to the 2017 ILAE seizure-type classification (Fisher et al., 2017). The Engel classification was used to categorize post-surgical outcome in children who had surgery at our institution following inpatient MEG. In addition, MEG’s contribution to decision making was judged and classified in six distinct categories, as presented in Table 1.

**TABLE 1:**
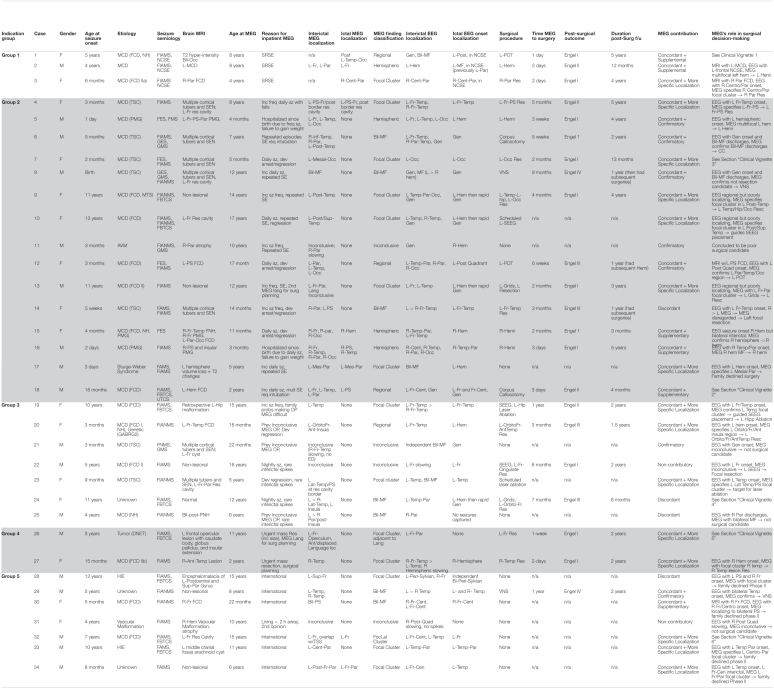
Findings and characteristics of subjects. (Followed by the already existing explanation of abbreviations).

*MCD, malformation of cortical development; FCD, focal cortical dysplasia; NH, gray matter heterotopia; TSC, tuberous sclerosis; PMG, polymicrogyria; MTS, mesial temporal sclerosis; SEN, subependymal nodules; FAMS, focal aware motor seizures; FIAMS, focal impaired awareness motor seizures; FMS; focal motor seizures; FES, focal epileptic spasms; FNMS, focal non-motor seizures; FIANMS, focal impaired awareness non-motor seizures; FBTCS, focal to bilateral tonic-clonic seizures; GES; generalized epileptic spasms; GMS, generalized motor seizures; SE, status epilepticus; NCSE, non-convulsive status epilepticus; SRSE, super-refractory status epilepticus; UTCS, unknown onset tonic-clonic seizures; TSS, Tactile Somato-Sensory Stimulation; res, residual; Inc, increasing; mult, multiple; req, requiring; sz, seizure(s); dev, developmental; lang, language; surg, surgical; prev, previous; probs, problems; OP, outpatient; VNS, Vagus Nerve Stimulator; MF, multifocal; L, left; R, right; Hem, hemisphere; Cent, central; Ant, anterior; Post, posterior; Sup, superior; Inf, inferior; Mid, middle; Mes, mesial; Lat, lateral; Bil, bilateral; PS, Peri-Sylvian; ED, epileptiform discharge; loc, localization; Hip, hippocampal; Fr, frontal; Temp, temporal; Par, parietal; Occ, occipital; Gen, generalized; POT, parieto-occipito-temporal disconnection or lobectomy; Res, resection; Hemi, Hemispherotomy; SEEG, stereoelectroencephalography; n/a, not applicable.*

MEG recordings were performed in accordance with the ACMEGS clinical practice guidelines (Bagić et al., 2011). All studies were conducted in the supine position inside a magnetically shielded room, using a 306-channel whole head MEG system (Triux, MEGIN, previously known as Elekta-Neuromag, Helsinki). Simultaneous EEG was recorded using a 22-channel electrode array already placed in the hospital setting [pediatric intensive care unit (ICU) or epilepsy monitoring unit (EMU)], except in one case where only pre-surgical functional mapping was performed without EEG electrodes. Before recording, the positions of three external fiduciary points, five head position indicator coils, all EEG electrodes, and several hundred head shape points were obtained (Mosher and Funke, 2020). During all recordings, continuous head position monitoring was performed to account for any shift in head position. Acquisition parameters for MEG/EEG included a sampling rate of 1,000 Hz and a band-pass filter of 0.1 to 330 Hz. Raw data was subsequently processed using a spatio-temporal signal space separation (tSSS) method (Taulu and Simola, 2006; Taulu and Hari, 2009; Nenonen et al., 2012) that compensates for significant head movements and suppresses interferences from nearby magnetic sources. That approach allows having operational medical equipment inside the shielded room, like a ventilator, and still obtaining data of highly satisfactory quality for analysis. Data processing and analysis were carried out using the vendor software packages (DANA and MaxFilter by MEGIN, Helsinki). Source analysis was computed using the multiple Equivalent Current Dipole (ECD) model. The MEG and EEG data were visually inspected for focal slowing and epileptiform discharges by a qualified MEG reader and an epileptologist. Spikes used for dipole fitting and coregistered to the patient’s brain MRI were retained if statistically significant (reduced Chi square >1.0 and <2.0, confidence volume ≤1000 mm^3^, source strength 100–500 nAm, goodness of fit > 80%). Language mapping, and source estimation was assessed according ACMEGS clinical practice guidelines for presurgical functional mapping (Breier et al., 2001; Papanicolaou et al., 2004; Burgess et al., 2011).

## Results

A total of 816 MEG studies were performed from 2012 to 2020, of which 478 (59%) were on children between 3 months and 18 years of age. Of this group, 444 (93%) of the studies were done as outpatient, and the remaining 34 (7%) were performed during hospitalization, as inpatient studies.

### Characteristics of Subjects

**FIGURE 1:**
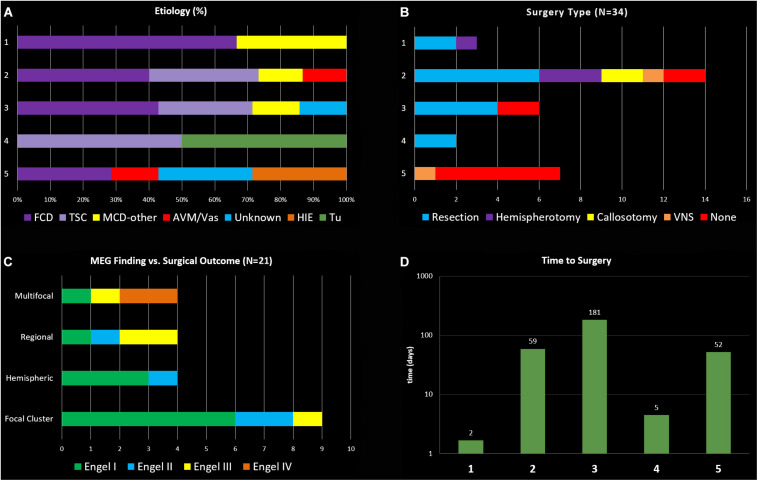
**(A)** General etiology types for the 5 clinical indication groups (FCD, focal cortical dysplasia; TSC, tuberous sclerosis complex; MCD, migration disorder of cortical development; AVM, arterio-venous malformation, Vas vascular; HIE, hypoxic-ischemic encephalopathy; Tu, tumor). **(B)** Distribution of surgical interventions in the five groups. **(C)** Main MEG findings and respective surgical outcomes. **(D)** Logarithmic display of days between MEG recording and surgical intervention in the groups.

Overall, there were 16 females and 18 males, with a mean age of 7.3 years at the time of inpatient MEG. The most common etiology was malformation of cortical development (MCD) in 25 children (74%). Specific types of MCD findings, identified by imaging, pathology, or both, were focal cortical dysplasia (FCD) in 14 (41%), Tuberous Sclerosis Complex (TSC) in 7 (21%), polymicrogyria (PMG) in 3 (9%), and nodular grey matter heterotopia (NH) in 4 (11%), with some patients having multiple types. Three children (9%) had a vascular malformation, 2 children (6%) had hypoxic Ischemic encephalopathy from birth, 1 child (3%) had a tumor (DNET), and in 3 children (9%) etiology was unknown (see Figure 1). Age of seizure onset ranged from the first day of life to 13 years old, with a mean age of 3.6 years old. All subjects had at least one focal seizure type at the time of MEG, and 20 (59%) had multiple seizure types, with the most common seizure types being focal impaired-awareness motor seizures in 22 (65%) and then focal impaired-awareness non-motor seizures in 9 (26%). Four children had focal epileptic spasms and 2 had generalized epileptic spasms, and 3 children had non-convulsive status epilepticus at the time of MEG. Brain 3T (or 1.5T in patients with VNS) MRI findings were abnormal in 28 children (82%), with 18 (53%) having a focal or hemispheric abnormality, 10 (29%) with multi-lesional findings; 6 (18%) had non-lesional or normal MRIs. Five children (15%) had a history of prior brain surgery at the time of MEG.

In terms of MEG findings, interictal epileptiform localization showed a single focal cluster in 16 (47%), scattered regional in 4 (12%), scattered hemispheric in 4 (12%), bilateral multifocal in 6 (17%), and inconclusive in 4 (12%) (see Figure 1). Ictal MEG data was obtained in 9 children (26%). All children had tactile somatosensory stimulation (TSS) localization performed (Mertens and Lütkenhöner, 2000) unless it was not possible due to too-frequent epileptiform activity, and one child had language lateralization performed. 21 children (62%) had subsequent neurosurgical intervention at our institution, of which 11 had resection, 4 had hemispherotomy, 2 had parietal-occipital-temporal lobectomies or disconnection (POT), 2 had corpus callosotomy, 1 had VNS (vagus nerve stimulator) placement, and 1 had laser ablation (see Figure 1). Two of these children underwent subsequent SEEG evaluation before laser ablation and resection, respectively; one child had subdural grid electrode evaluation before resection. Additionally, one child has laser ablation currently scheduled but not yet performed, and one child has SEEG evaluation scheduled but not yet performed. For those patients who had surgery subsequent to inpatient MEG, time from inpatient MEG to surgery ranged from 1 day to 1 year, with an average of 3 months. Duration of post-surgical follow-up ranged from 3 months to 5 years, with an average follow-up duration of 2.4 years (see Figure 1).

The reasons for performing inpatient MEG were determined by review of each subject’s available medical records, and five distinct indications were identified by consensus of the investigators. Relevant data for each case is presented in Table 1, with cases grouped by indication. What follows is a definition of each indication, with an illustrative case vignette (2 vignettes for Indication 2), and further analysis of each indication group, including case characteristics, MEG and EEG findings, surgical treatments and outcomes.

The label *Concordant*+*supplemental* applies to six patients, *Concordant*+*more specific localization* applies to 15, *Concordant*+*confirmatory* applies to five, *Confirmatory* applies to two, *Discordant* applies to four, and *Non-contributory* applies to two.

### Indication 1: Patients in Super-Refractory Status Epilepticus (SRSE) With Focal or Lateralized EEG

Three children had inpatient MEG due to ongoing pharmaco-resistant non-convulsive status epilepticus on continuous EEG monitoring. These children all had known intractable focal epilepsy, and due to an ongoing episode of SRSE, were being considered for emergent surgical intervention. In these cases, all had malformations of cortical development detectable on MRI, and focal or at least lateralized seizure activity on EEG. MEG results provided crucial localization data used in formulating a decisive plan for emergent surgical intervention that resulted in resolution of the SRSE in all three cases.

#### Clinical Vignette 1

**FIGURE 2:**
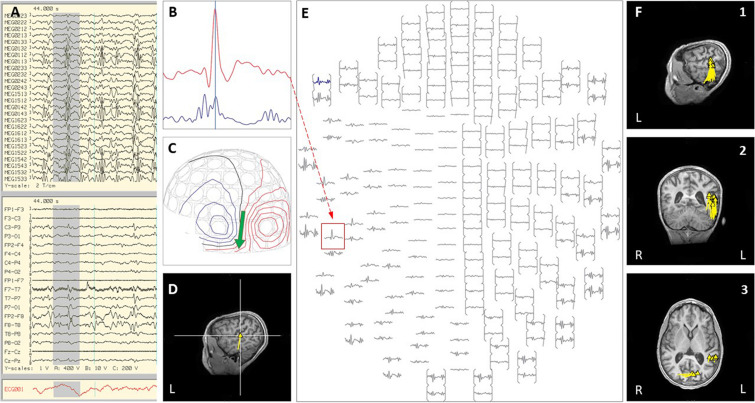
Case 1 **(A–E)** an example of an individual simultaneous MEG–EEG discharge in the left temporal sensor area. **(B)** Selected MEG channel and time instance of the magnetic field distribution **(C)** in sensor space with the projected source estimate (green arrow). The sagittal MRI slices **(D)** indicates the origin and source orientation of a single discharge at the left temporo-parietal junction. The MEG channel plot **(E)** of the selected time interval as shown in **(A)** shows the region of interest and the planar gradiometer channel (red square) with the earliest peak time. The summary pictures **(F1–F3)** give an impression of the epileptiform activity before surgery (yellow triangles).

Case 1 was born at 28-weeks-gestation and had normal development until 5-years of age, when she developed intractable epilepsy with multiple seizure types including generalized tonic-clonic seizures, focal motor seizures, and head drop seizures, repeated episodes of status epilepticus, global developmental regression and progressive ataxia. Epilepsy evaluation revealed bi-frontal interictal epileptiform discharges and generalized or left-hemispheric seizure onset. High resolution epilepsy protocol MRI brain showed T2 white matter signal abnormality around the occipital horns extending to the subcortical white matter. A corpus callosotomy was performed, and immediately following the procedure she was noted to be in non-convulsive status epilepticus on EEG, with epileptiform discharges exhibiting a left hemisphere or left posterior quadrant predominance. All attempts to abort the activity with pharmacological treatment proved unsuccessful over multiple days. As she fit criteria for super-refractory status epilepticus and had lateralized EEG activity, an inpatient MEG was done emergently to explore the option of abortive epilepsy surgery. Performing the MEG evaluation required substantial coordinated planning, including use of a portable mechanical ventilator, supportive care from a respiratory therapist, PICU nurse, pediatric neurology physician, and two MEG technicians (see Figure 1). MEG ictal data was successfully recorded, and was localized to the left superior temporal-parietal region and the left occipital region independently (see Figure 2). A left parieto-occipital-temporal lobectomy (POT) with intraoperative ECoG was performed the next day, with resolution of status epilepticus. She remained seizure free for 5 years except for rare self-limited seizures due to medication non-compliance. Surgical pathology revealed non-classified focal cortical dysplasia and nodular heterotopia, and genetic evaluation revealed SCN8A. Unfortunately, the child subsequently succumbed to aspiration pneumonia at age 13. The use of inpatient MEG in this case helped formulate and expedite a surgical plan that was critical in the resolution of her SRSE, which not only prevented further complications but was likely a life-saving event, and resulted in markedly improved quality of life.

### Indication 2: Patients With Intractable Epilepsy With Frequent Electroclinical Seizures, and/or Frequent or Repeated Episodes of Status Epilepticus

Fifteen children (44%) had inpatient MEG performed under this indication. When children with intractable epilepsy develop seizures that occur daily or become life-threatening, the usual pace of outpatient data gathering is sometimes not adequate to meet the needs of a more urgent pre-surgical evaluation. Frequent hospitalizations due to seizure exacerbations or prolonged seizures can result in missed outpatient testing appointments, or make outpatient testing unsafe or impractical. In these instances, performing the MEG during inpatient EMU admission provides a more reliable and safe way of completing the evaluation and often results in ictal MEG data acquisition, as it did in 5 children in this group (33%). Ictal localization on MEG is uncommon in most patients, but can vastly increase the helpfulness of MEG in identifying the seizure focus. The ability to gather all data in the pre-surgical evaluation as quickly as possible can also help to more efficiently finalize a plan for surgical intervention, and MEG localization can be invaluable in more precise surgical planning. Ictal MEG data can be helpful in obviating the need for invasive monitoring prior to definitive surgical intervention, be it laser ablation, resection or disconnection, corpus callosotomy, or hemispherotomy. In children with this indication, more timely surgical intervention can be life-saving, or result in vastly improved safety, quality of life, and/or developmental outcome.

#### Indication 2, Clinical Vignette 2

**FIGURE 3:**
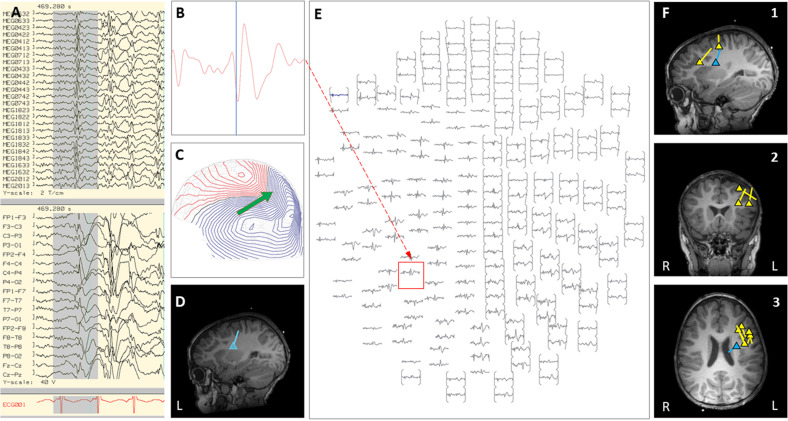
Case 18 **(A–E)** an example of an individual simultaneous MEG–EEG seizure onset in the left intra-Sylvian area. **(B)** Selected MEG channel and time instance of the magnetic field distribution **(C)** in sensor space with the projected source estimate (green arrow). The sagittal MRI slices **(D)** indicates the origin and source orientation of the seizure onset. The MEG channel plot **(E)** of the selected time interval as shown in **(A)** shows the region of interest and the planar gradiometer channel (red square) with the earliest peak time. The summary pictures **(F1–F3)** give an impression of the epileptiform activity before surgery (yellow triangles) and seizure onset (blue triangle).

Case 18 is a boy who was born at full-term with normal development and seizure onset at 18 months of age. Over the next 6 months, his epilepsy failed to respond to multiple trials of AEDs, and he started to have daily prolonged seizures and increasingly frequent episodes of status epilepticus requiring hospitalization and intubation. Seizures consisted of staring, variable head turning to either side, often followed by right greater than left clonic activity, or abrupt onset generalized clonic activity. Brain MRI showed subtle diffuse abnormal gyration and blurring of gray-white junction in multiple regions of the left hemisphere. Due to his frequent hospitalizations, outpatient MEG appointments were missed. Of note, a half-brother had undergone epilepsy surgery for intractable epilepsy as a child, which was initially successful, however, he later died from SUDEP. During an exacerbation with increased seizure frequency, the patient was admitted to the EMU. Video EEG showed frequent interictal epileptiform discharges in the left frontal and left centro-parietal region, with multiple seizures captured with left hemispheric or generalized onset. An inpatient MEG was performed and interictal and ictal epileptiform activity showed a scattered localization distributed over the left frontal and central regions (see Figure 3). Given this broad distribution of MEG dipoles over the left hemisphere, the degree of clinical acuity associated with his current admission, and parent choice after thorough discussion of all treatment options, the decision was made to proceed with a corpus callosotomy. This procedure was completed during the admission, 3 days after the inpatient MEG. Post-operatively, during the first 4 months of follow-up, the child continues to have focal motor seizures with impaired awareness, but with a 50–75% improvement in frequency and seizure duration. There have been no further convulsive generalized seizures or episodes of status epilepticus.

In this clinical scenario, utilization of inpatient MEG helped to expedite the presurgical workup for this patient with intractable epilepsy who was experiencing daily seizures and repeated episodes of status epilepticus. Completion of the MEG as an inpatient allowed a more clear understanding of likelihood of success and risks of the various options for surgical intervention, while also providing a practical solution that allowed the patient to experience a notable improvement in overall seizure burden and consequently a significant improvement in overall quality of life. In less urgent circumstances, this MEG result would most likely have led our group to pursue a phase II evaluation with left hemispheric SEEG. However, given the risks of prolonging the time to definitive intervention, a corpus callosotomy was chosen as more appropriate, and the MEG provided crucial insight that eliminated the possibility of focal resection without phase II evaluation.

#### Indication 2, Clinical Vignette 3

**FIGURE 4:**
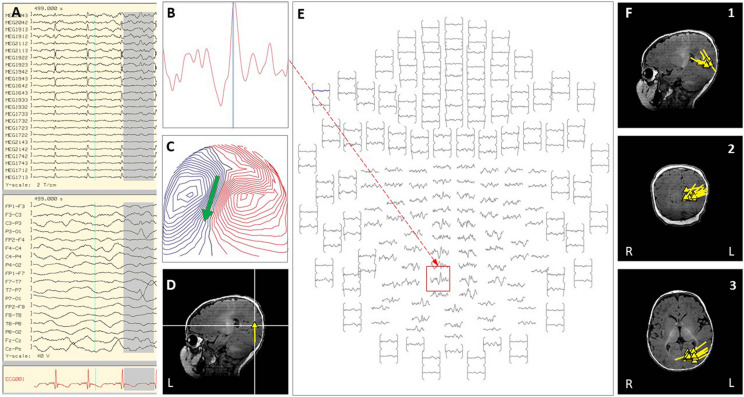
Case 7 **(A–E)** provide an example of an individual simultaneous MEG–EEG seizure onset in the left interhemispheric area. **(B)** Selected MEG channel and time instance of the magnetic field distribution **(C)** in sensor space with the projected source estimate (back of the head, green arrow). The sagittal MRI slices **(D)** indicates the origin (triangle) and source orientation (tail) of the spike discharge. The MEG channel plot **(E)** of the selected time interval as shown in **(A)** shows the region of interest and the planar gradiometer channel (red squared box) with the earliest peak time. The summary pictures **(F1–F3)** give an impression of the epileptiform activity (yellow triangles) originating all from the left mesial occipital cortex.

Case 7 is a girl born at full-term gestation with tuberous sclerosis complex and polycystic kidney disease caused by a deletion in 16p encompassing the TSC2 and PDK1 genes. Brain MRI revealed multiple cortical and subcortical tubers in both hemispheres and bilateral subependymal nodules. Epilepsy was diagnosed at 2 months of age when left occipital electrographic seizures with clinical correlate of intermittent eye deviation were detected on a screening scalp VEEG. These seizures continued despite multiple AED trials, and when she developed daily right-sided focal motor seizures with impaired awareness, focal epileptic spasms, and began demonstrating limitations in visual tracking and language development at 5 months of age, she was admitted to the EMU for pre-surgical evaluation. Video EEG captured multiple interictal epileptiform discharges in the left occipital region, with multiple seizures involving a left occipital onset and spread over the left posterior quadrant. Additionally, several episodes of focal epileptic spasms were captured arising from the left posterior quadrant. Due to the increasing frequency and severity of her seizures and onset of epileptic spasms, inpatient MEG was obtained during the admission. This revealed a focal cluster of interictal activity and seizure onsets in the left occipital region (see Figure 4). Focal resection was recommended in Case Management Conference, and parents finally agreed to proceed 2 months following the MEG, after failure of an additional AED (Vigabatrin). Left occipital resection of a cluster of cortical tubers corresponding to the MEG cluster was performed with electrocorticography and intra-operative Stealth MRI visualization of the MEG dipoles. The child has been seizure-free for the 13 months since surgery, with rapid improvement in all aspects of development, including return of visual responsiveness.

### Indication 3: Patients With Infrequent Epileptiform Discharges on EEG or Outpatient MEG, Who Require Holding or Reducing AEDs, or Other Special Circumstances Necessitating Inpatient Monitoring for Successful and Safe MEG Data Acquisition

Magnetoencephalography was performed as an inpatient due to indication 3 in 7 of our cases (21%). Six of these children had either a previously inconclusive outpatient MEG (3), or demonstrated infrequent interictal epileptiform abnormalities on their EEGs (3). For two of the children with rare interictal abnormalities, seizures only occurred at night. Performing these MEGs in an inpatient setting allowed for more control of the conditions, such as reversing sleep-wake cycle, monitored sleep-deprivation, and reducing or holding AEDs in a safe and monitored environment. In the remaining case (case #19), social factors made outpatient preparation for testing too unpredictable, and so testing in an inpatient environment allowed for more optimal preparation of the patient.

#### Indication 3 – Clinical Vignette 4

**FIGURE 5:**
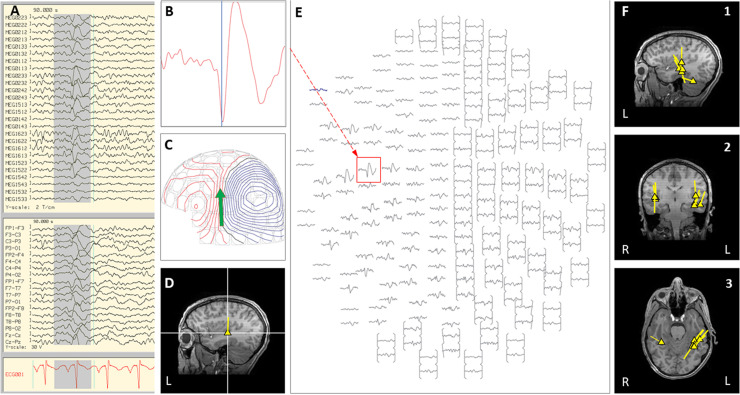
Case 24 **(A–E)** provide an example of an individual simultaneous MEG–EEG discharge in the left temporal sensor area. **(B)** Selected MEG channel and time instance of the magnetic field distribution **(C)** in sensor space with the projected source estimate (green arrow). The sagittal MRI slices **(D)** indicates the origin (triangle) and source orientation (tail) of a single discharge at the left temporo-parietal junction. The MEG channel plot **(E)** of the selected time interval as shown in **(A)** shows the region of interest and the planar gradiometer channel (red squared box) with the earliest peak time. The summary pictures **(F1–F3)** show left greater than right posterior temporal distribution (see Figure 5) (yellow triangles).

Case 24 was adopted at 3 years of age and had unknown birth, neonatal, and early developmental history, as well as mild intellectual disability. Seizure onset was at 11 years old, with nightly nocturnal focal seizures with impaired awareness, head and body turning to the right and automatisms, which proved unresponsive to multiple AEDs. On prolonged scalp video EEG, rare interictal epileptiform discharges were seen from the left temporal region, with ictal onset that appeared generalized. Brain MRI was normal, and outpatient MEG was inconclusive due to lack of interictal discharges. MEG language testing (as an outpatient) revealed bilateral posterior temporal and inferior frontal involvement in receptive language processing. PET demonstrated hypometabolism in the left inferolateral temporal lobe. In order to obtain SPECT and maximize yield of MEG, she was admitted to EMU, antiepileptic medications were reduced, and the sleep-wake cycle was reversed using sleep deprivation. Ictal SPECT revealed increased uptake in the left frontal lobe and left insula with a lesser extent of uptake in the left temporal lobe. On the third day of admission, an inpatient MEG was done with the child asleep, and only interictal data were captured, with 70% of discharges localizing to the lateral temporal and posterior insular region, and 30% of discharges localizing to the right posterior temporal region (see Figure 5). Due to the discordant but left hemispheric predominant data and concern about language localization given bilateral MEG language testing results, the plan from case management conference was to proceed with a Phase II invasive evaluation with subdural grid electrodes. Seven months following inpatient MEG, left subdural grid evaluation revealed seizure onset over the left orbitofrontal region with rapid spread to the left posterior temporal region, and left orbitofrontal resection was performed. In the 8 months post-surgery, the patient has had worthwhile improvement with >50% reduction in seizure burden.

### Indication 4: Patients Requiring MEG Mapping of Eloquent Cortex or Interictal Spike Localization in the Setting of Tumor Resection or Other Urgent Neurosurgical Intervention

For clinical situations in which urgent inpatient neurosurgical intervention is felt to be necessary, MEG can be performed pre-operatively as an inpatient for interictal localization or functional mapping to help guide surgical planning and decision-making. In our 2 cases, both were patients with epilepsy who were admitted when MRI detected an intracranial mass of unknown etiology, for urgent resection in the setting of a suspected malignancy.

#### Indication 4 – Clinical Vignette 5

**FIGURE 6:**
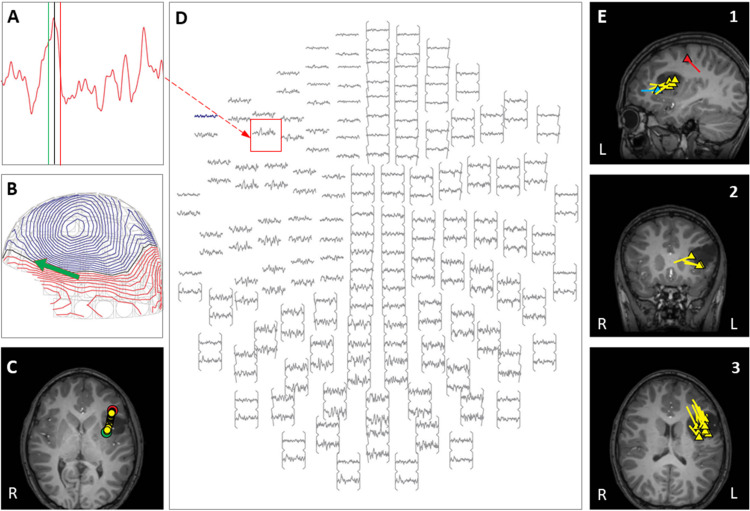
Case 26 **(A–E)** provide an example of left-sided language activation in the Broca’s area, using a silent verb generation task. **(A)** Left frontal MEG channel with clear activation pattern between green and red cursor from 425 to 455 ms post stimulus, **(B)** MEG map at 440 ms post stimulus (black cursor position in **A**) with the projected source estimate (green arrow), **(C)** axial MRI with plotted source origins (round dots) from 425 ms (green) to 455 ms (red), **(D)** in sensor space with boxed channel (red) as seen in **(A)**. **(E1–E3)** The summary pictures demonstrating epileptiform activity (yellow triangles), language activation (blue triangle), and tactile somatosensory activation (red-triangle). Of note, epileptiform discharges appear posterior to the language activation area.

Case 26 was an 11-year-old boy with epilepsy characterized by focal motor seizures with impaired awareness and auditory aura, with subsequent tonic-clonic generalization. Initial brain MRI had revealed a lesion in the left frontal operculum, and VEEG revealed left frontoparietal interictal epileptiform discharges. Epilepsy was initially well-controlled with AEDs, but the child was admitted when seizures became suddenly refractory to medications, and a follow-up MRI brain revealed interval growth of the lesion with caudate body, globus pallidus, and insular extension. Urgent neurosurgical intervention was recommended due to change in size of the mass and recent conversion to intractable epilepsy. Inpatient MEG was performed to localize interictal epileptiform activity and lateralize functional language activity, in relation to the mass. Receptive language processing was left hemispheric involving posterior and superior temporal and inferior parietal cortex. In an additional non-standard step, as seen in Figure 6, expressive language function was localized anterior to the interictal epileptiform dipoles and ventral to the mass lesion. This mapping allowed for a higher level of confidence that surgical resection of the entire anatomic lesion could be done with minimal risk of language deficit. One week later, surgical resection of the lesion was performed, and histopathology revealed a WHO Grade 1 dysembryoplastic neuroepithelial tumor (DNET). Post-resection, the patient experienced no neurological deficits, and 2 years post-resection remains seizure-free and has been weaned off of all antiepileptic medications.

### Indication 5: International or Long-Distance Patients, Where Outpatient MEG Is Not Possible or Practical

In 7 cases (21%), MEG was performed as an inpatient because the patient was traveling for evaluation from long distances, or internationally. In these cases, if there is no difference in reimbursement for services or cost to the family, the goal should be to make the evaluation as efficient, practical, and convenient as possible for the patient and their family.

#### Indication 5 – Clinical Vignette 6

**FIGURE 7:**
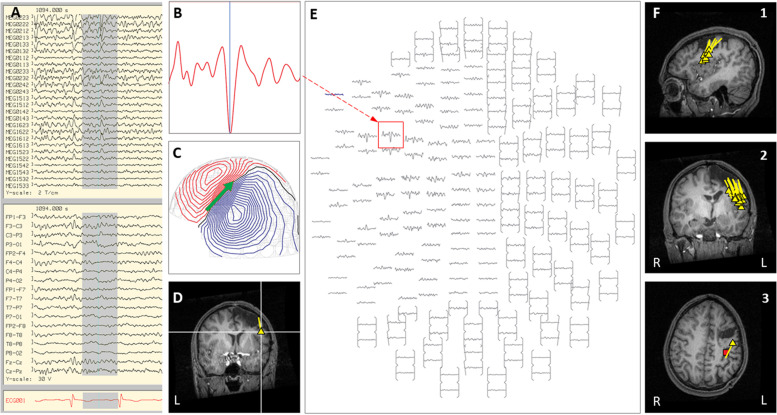
Case 32 **(A–E)** provide an example of an individual simultaneous MEG–EEG seizure onset in the left intra-Sylvian area. **(B)** Selected MEG channel and time instance of the magnetic field distribution **(C)** in sensor space with the projected source estimate (green arrow). The coronal MRI slices **(D)** indicates the origin (triangle) and source orientation (tail) of the discharge (highlighted in **A**). The MEG channel plot **(E)** of the selected time interval as shown in **(A)** shows the region of interest and the planar gradiometer channel (red square) with the earliest peak time. The summary pictures **(F1–F3)** gives an impression of the epileptiform activity before surgery (yellow triangles) originating from an area inferior to the prior laser-ablation cavity. The red square marks the source of the tactile somatosensory response.

Case 32 is a 15-year-old boy from Mexico, traveling to our institution for a second opinion regarding persistent focal seizures despite multiple surgical interventions. His seizure onset was at 7 years of age, with focal motor seizures with impaired awareness that became medically intractable; a pre-operative brain MRI had revealed focal cortical dysplasia of the left frontal lobe. Laser ablation of the left premotor area had been performed at 10 years of age, then left-sided subdural grids and subsequent left craniotomy for premotor and supplementary motor area resection at 11 years of age, followed by VNS placement at 13 years of age. As he was traveling from another country and was self-pay, all tests were performed as an inpatient as this made his evaluation faster and more efficient, with no change of cost for the family. In the EMU, VEEG revealed interictal epileptiform discharges that were left frontocentral and left temporal, as well as focal motor seizures with impaired awareness arising from the left frontal region. Inpatient MEG showed frequent ictal and interictal activity arising from the left frontal lobe, which overlapped with localization of tactile somato-sensory stimulation (see Figure 7). The family was counseled that seizure onset zone and primary motor cortex were likely overlapping, and that further resection would carry significant risk of right hand fine motor deficits. We also discussed SEEG evaluation for possible RNS (responsive neurostimulator) placement as another option. Family chose to start ketogenic diet upon return to Mexico.

## Discussion

### Super-Refractory Status Epilepticus in Children

The most important and highest acuity indication in our cohort of cases was Indication 1: patients in super-refractory status epilepticus (SRSE) with focal or lateralized EEG. Status epilepticus (SE) is a common neurological emergency in both adults and children, with an annual incidence of 10–40 cases per 100,000 population (Abend and Loddenkemper, 2014; Betjemann and Lowenstein, 2015) and a mortality rate that remains high, depending on the underlying etiology (Abend and Loddenkemper, 2014; Kantanen et al., 2015; Sánchez and Rincon, 2016). The classification of SE into convulsive or non-convulsive according to clinical semiology, and refractory SE (RSE) or super-refractory SE (SRSE) according to seizure duration, as outlined by the International League Against Epilepsy (ILAE) (Trinka et al., 2015), seeks to promote early recognition in clinical practice and guide therapeutic approaches. The mortality rate for children with SRSE was 19–43% in retrospective case studies (Gilbert et al., 1999; Kim et al., 2001; Sahin et al., 2001; Fernández et al., 2014). The current management of patients diagnosed with SRSE, defined as continuous or recurrent seizures lasting 24 h or more following initiation of anesthetic medications (Cuero and Varelas, 2015), derives from a number of case reports and expert opinions on pharmacologic and non-pharmacologic treatments, with varying success results (Hocker et al., 2014; Bayrlee et al., 2015; Glauser et al., 2016). The therapeutic choice remains individualized, based on the suspected etiology as well as the results of laboratory, neurophysiologic, and neuroimaging investigations. In cases of SRSE, scalp EEG data alone may be insufficient to achieve a precise and accurate localization of the epileptogenic zone. In addition, adequate recording can be hampered by technical difficulties that arise in the intensive care setting (Friedman et al., 2009). A report of the American Academy of Neurology found that epileptiform abnormalities occurred in only 43% of EEGs in children with SE (Riviello et al., 2006). The same review identified a mere 8% of children with SE showing abnormalities on neuroimaging. Another prospective study on 144 children presenting with SE found abnormal EEG results in 60% of cases, with 19% having focality. In this study, neuroimaging (CT and/or MRI) revealed a diagnostic etiology in 30% of cases (Singh et al., 2010). Overall, the precipitating etiology remains undetermined in a significant number of patients presenting with SE (Riviello et al., 2006; Novy et al., 2010; Singh et al., 2010), and consequently, they are classified as cryptogenic or idiopathic. Interestingly, all of our cases with SRSE had abnormal MRIs, and were found to have various malformations of cortical development retrospectively, on pathology.

### Considerations Related to MEG Recording and Data Quality in ICU Setting

In situations where pharmacologic treatments have failed in SRSE, and the EEG, semiology, or imaging suggests a potential underlying epileptic focality, considering neurosurgical treatment options is warranted (Alexopoulos et al., 2005). In our institutional experience, MEG can contribute significantly to making this therapeutic decision. There are a number of advantages to performing an inpatient MEG in these unstable patients. Firstly, MEG is a non-invasive study that can provide valuable information in a short period of time, as was seen in Case 1, with a total recording time of 7 min. In addition, MEG has extensive spatial resolution that far exceeds that obtained by scalp EEG alone in the intensive care setting. It also has very high temporal resolution, capable of detecting rapidly changing patterns of brain activity. This can be crucial when seizures are frequent and repeated, such as in the various stages of SE.

However, there are a number of potential limitations to performing an inpatient MEG in unstable patients with SRSE, which must be taken into consideration. At present, in order for the infinitesimally small magnetic fields that are associated with epileptic discharges to be detected, MEG recordings must still be performed within a magnetically shielded room that deflects the external magnetic noise of the surrounding environment. These rooms can often be located at a distance from the intensive care unit, and it may require a coordinated team of physicians, nurses and technicians to plan and carry out successful transportation of the patient. Furthermore, a number of devices will typically accompany any patient requiring intensive care, including portable ventilators and other monitors that can be the source of considerable competing magnetic noise. Such artifacts must be adequately processed during and after recordings with the use of the tSSS method (Burgess, 2020; Mosher and Funke, 2020). The cases presented in this paper do, however, demonstrate that such technical aspects can be overcome, and good quality data can be obtained in critically ill patients in an acute, inpatient setting.

**FIGURE 8:**
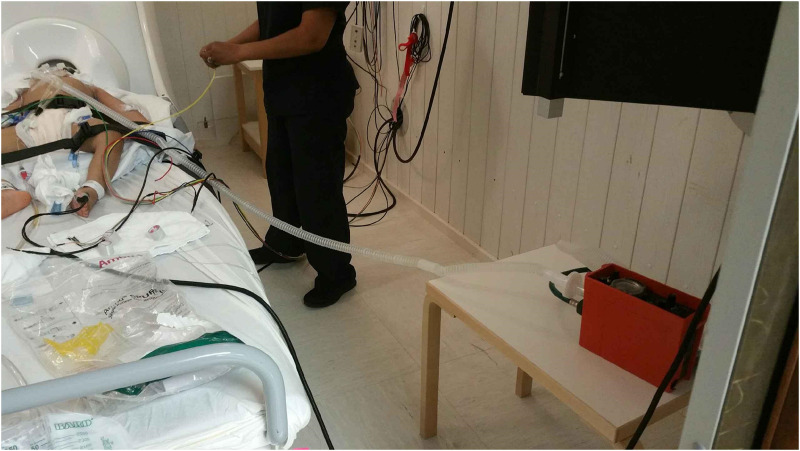
If medical equipment is placed a sufficient distance from the helmet, then its artifacts may be reduced to an acceptable level, such that it could be removed in signal processing. Shown here is a pediatric intensive care unit (PICU) patient during an MEG examination with an MRI-compatible ParaPAC ventilator positioned in the corner of the magnetically shielded room (red box at the lower right). It is positioned as far as possible away from the patient and the sensor helmet. The separate side table minimizes vibration artifacts. For situations where anesthesia of a patient is needed, then the suction, oxygen, intravenous lines, pulse oximetry fiber optics cable, electrocardiogram (ECG) leads, etc. can generally be brought through port tubes in the walls of the room.

The preparation protocol for inpatient MEG recording in these three cases (see section “Indication 1”) was extensive, involving a step-by-step approach from the intensive care unit to the MEG recording room. As with all mobilization procedures of patients from the intensive care unit, patient transport relied on effective team communication and extensive pre-planning. To ensure safety during transport and MEG recording, the patients were transitioned from an in-room mechanical ventilator to a portable mechanical ventilator with a care team consisting of a Pediatric Transport Nurse, the Pediatric Neurologist on service, the MEG Technologist, and a Respiratory Therapist. The patient was maintained on portable mechanical ventilator support throughout the duration of MEG recording, with the care team present at bedside. The set-up involved in the magnetically shielded room is illustrated in Figure 8. To avoid burst-suppression activity throughout the MEG recording, a pentobarbital-induced coma in all three patients was temporarily put on hold on the morning of the exam day. The data obtained was then reviewed and presented at our Pediatric Epilepsy Surgery Conference to develop an appropriate surgical plan.

Surgical management of SRSE has been successful when presurgical evaluation has identified a focal onset to seizures (Cuello-Oderiz et al., 2015; Basha et al., 2017), achieving both SRSE cessation and long-term seizure freedom. A thorough evaluation should therefore promptly be considered in such patients. When available, MEG represents a supplementary non-invasive testing modality that can provide vital localization data regarding ictal onset in unstable patients, as well as in states of continuous or repetitive seizure activity. In our cases, MEG was followed by rapid surgical intervention and cessation of SE was obtained in all three cases after surgery. The contribution of MEG was substantial, as EEG was lateralizing but non-localizing in all three cases. MEG identified the most active epileptogenic area and provided additional information that had a direct effect on surgical decision-making. The utilization of inpatient MEG can thus prove helpful in identifying a focal onset of epileptiform activity, and subsequently guide urgent surgical intervention in patients with SRSE.

### MEG in Children With Very Frequent Seizures

In children with severe intractable or catastrophic epilepsy (see section “Indication 2”), seizure frequency can be quite high, sometimes with many seizures occurring daily. In some patients, there is also tendency toward prolonged seizures or repeated episodes of status epilepticus, leading to frequent hospitalizations. Epilepsy in these children has severe psychological and social consequences, which imparts an increased risk of bodily injury, systemic complications, and SUDEP, all contributing to increased cumulative mortality (Laxer et al., 2014) and morbidity. Overall, patients with childhood-onset epilepsy have a higher rate of mortality than the general population. 2,239 subjects from four different pediatric cohorts with childhood-onset epilepsy were followed for roughly 30,000 person-years and found to have a mortality rate of 228 per 100,000 person-years. In comparison to age-matched controls in the general population, this rate was 5-10 times higher (Berg et al., 2004a, 2013). Though all-cause mortality is more associated with non-epilepsy related complications, certain characteristics such as a history of status epilepticus and a diagnosis of refractory epilepsy increases the overall risk (Laxer et al., 2014; Donner et al., 2017; Smith and Beckerman, 2020).

In addition to the effect on mortality, frequent clinical or subclinical seizure activity can also have a negative impact on development and cognitive performance in children (Bulteau et al., 2000; Bjørnaes et al., 2001; Berg et al., 2004b), which often correlates with the severity and frequency of seizures. Neuropsychological impairment is a recognized comorbidity in patients with epilepsy and is influenced by several factors including age of seizure onset, seizure type, seizure frequency, seizure duration, and anti-epileptic medications. In patients with intractable epilepsy, persistent interictal and ictal activity through a critical period of development disrupts neuronal networks, neurogenesis, metabolic homeostasis, and brain architecture, all of which can interfere with cognitive function and contribute to neurocognitive deficits of varying degrees (Holmes, 2004; Chapman et al., 2015; Nickels et al., 2016).

For children with intractable epilepsy who have frequent seizures or repeated episodes of SE, the impact on developmental and cognitive outcome and their high risk of morbidity and mortality adds additional urgency to completing pre-surgical evaluation and determining candidacy for epilepsy surgery. This indication was the most common one for inpatient MEG in our cohort, comprising 44% of the total. For these children, performing MEG on an inpatient basis expedited obtaining the critical mass of data required for decision-making regarding surgical candidacy and planning. Especially for children who are experiencing increased seizure frequency and unpredictable hospitalizations, inpatient testing avoids the delays and subsequent clinical deterioration that could occur during an outpatient workup.

Twelve of our 15 children in this indication group (#2) had epilepsy surgery, with time from inpatient MEG to surgery that ranged from 3 days to 8 months, and an average time to surgery of 2 months. We found that there were a variety of factors that contributed to this time lag, ranging from parental decision to further unexpected hospitalizations and scheduling delays (including those due to the COVID-19 pandemic). From the perspective of their high degree of intractability as a group, it is reassuring that 7 (58%) of the children were seizure-free after surgery, and that 75% had a surgical outcome of Engel class I or II.

### MEG in Children With Infrequent Interictal Epileptiform Discharges

Up to 26% of clinical MEG studies for epilepsy will have inconclusive results (Alkawadri et al., 2013). Although children tend to have more frequent epileptiform discharges than adults, there are still a proportion of children with intractable epilepsy who have infrequent epileptiform discharges under baseline conditions (i.e., taking AEDs and not sleep-deprived). In most cases, families can be instructed before an outpatient study to sleep-deprive the child, however, there are some children for whom this is difficult to achieve for a variety of reasons, or who still have an inconclusive outpatient MEG despite the best efforts of the family in this regard. At our institution, we do not perform MEG studies with sedation or general anesthesia, the exception being for Indication 1, as described above. Patient preparation involves sleep deprivation with an average of 20–30% reduction in normal hours of sleep, which depends on the cooperation of the family in the outpatient setting, and this important step can be more easily controlled in the inpatient setting when needed. Once in the MEG laboratory setting, scalp electrodes are put into place and a digitization of fiducial points, head position indicator coils, EEG electrode location, and sufficient head shape points are completed. Before entering the shielded room, our protocol for infants and young children often involves feeding the patient and placing in the supine position, wrapped in warm blankets and provided with additional gel cushioning to maintain the body axis in a neutral position and minimize lateral movement. The infant or child is then placed inside the shielded room accompanied only by the pediatric-specialized technician. Additional feeding and soothing techniques are carried out if the child appears distressed and until sleep is obtained. This preparation phase often takes about 30–45 min. Once in the MEG and asleep, the infant or child is safely fastened using padded belts and is horizontally advanced to ensure adequate head coverage, using the eyebrow line as the advance limit. Additional padding is placed to either side of the face to avoid harm and limit head movements, and in the occipital groove. Recording times are usually 60 min. We have found that using these techniques and performing the MEG without the use of sedation allows maximal spike capture and increases the likelihood of a sleep recording. Most of the time, with adequate communication from the technician, the family is able to follow our preparation protocols, but in the cases where this is not achievable, the inpatient environment allows better control of circumstances and protocol adherence. Counseling families to reduce or stop AEDs in children prior to outpatient MEG is not done at our institution, and is generally not advised, due to the risk in these children of status epilepticus and seizure safety concerns. Hence our Indication 3 for inpatient MEG, in patients with infrequent epileptiform discharges on EEG or inconclusive outpatient MEG, who require holding or reducing AEDs, or have other special circumstances such as difficulty with preparation techniques, necessitating inpatient monitoring for optimal and safe MEG data acquisition. In an inpatient setting, correct sleep deprivation protocols can be facilitated, which have been shown to increase cortical excitability and thus increase the frequency of interictal discharges and seizures (Samsonsen et al., 2016; Badawy et al., 2017). Additionally, AEDs can be reduced or held, thus increasing the likelihood of capturing interictal or ictal abnormalities in the safe and monitored environment of the EMU. In patients who have had an initial inconclusive MEG study, the yield of a repeat study is significant, especially when the repeat study can be done in better clinical circumstances for provoking interictal and ictal abnormalities. In our cohort, there were 7 cases falling under this indication. 3 of these had previously inconclusive MEGs as outpatient, 2 of which were successful when repeated as inpatient. The other 4 children had rare interictal spikes on EEG or circumstances making successful completion of the outpatient test difficult. Two children still had inconclusive MEG results with inpatient testing. Four of the 7 children went on to have epilepsy surgery at our institution, with an average time from MEG to surgery of 11 months, and only 2 of these had an outcome of Engel class II or better. 6 of the 7 had brain malformations, 2 of which were TSC, and one had a genetic mutation, GABRG2.

### Mapping of Eloquent Cortex With MEG

While the most frequent use of clinical MEG is for localization of epileptic foci in patients with intractable epilepsy, MEG is also used for pre-surgical mapping of various sensory modalities (Bagić and Burgess, 2020b). In children with epileptogenic lesions where malignancy is suspected, inpatient MEG can provide crucial information guiding urgent neurosurgical intervention. While this indication (#4) was a relatively infrequent one in our cohort, obtaining this MEG data quickly in an inpatient setting was the final and deciding piece of information that allowed optimal surgical resection in both of our cases, which occurred within a week of testing, with both patients having an Engel 1 outcome after 2 years.

### Practical and Logistic Considerations for Performing MEG

Although standard practice is to perform MEG as an outpatient test, the ease with which a MEG can be completed on an outpatient basis as part of a pre-surgical evaluation is heavily dependent upon the patient’s proximity to the MEG center, and to the referring Epilepsy Center. A recent Clinical MEG Survey indicated that there are currently 25 MEG centers in the United States that are considered to be actively engaged in providing significant clinical MEG services (Bagić and Burgess, 2020a). For those within the United States who do not live near one of these centers, an outpatient MEG evaluation can prove difficult or impossible from a social and logistical standpoint, and can even contributing to delays in care. In most countries outside the United States, clinical MEG centers are rare, and MEG may not even be accepted as standard of care for the evaluation of intractable epilepsy. For these reasons, it is not uncommon for Epilepsy Centers in the United States that have MEG Centers to see intractable epilepsy patients coming for pre-surgical evaluations from a distance of more than a 2-h drive, including those from out-of-state or international locations. Although financial sustainability of the MEG Center has to be a factor in designing each center’s practice guidelines and protocols, each center also has an ethical obligation to plan logistically efficient pre-surgical evaluations for those patients coming from a substantial distance. In many of these cases, such as those in our indication 5 group, inpatient MEG should be strongly considered, to avoid unnecessary social burdens or delays and to ensure that all necessary pre-surgical data, including MEG, is obtained. The method of reimbursement to the MEG Center should also be considered, as this may be different for international patients being evaluated in the United States, and may make the issue of differences in reimbursement for outpatient vs. inpatient settings moot.

### Contributions of MEG to Surgical Decision-Making

It is important to note that in our cohort of children in whom MEG was performed in the inpatient setting, only two children had MEG results that were non-contributory due to inconclusive data, and in one of these cases, the other testing modalities were also inconclusive, leading to the conclusion that the child was not a surgical candidate. In all the other cases, the MEG data contributed to the surgical decision-making process, even if surgery was not performed due to the family’s choice. In 15 cases (44%), the MEG provided data that was concordant with the other modalities of data, such as interictal and ictal EEG, semiology, MRI, PET, and SPECT, and provided unique information that was more specific in localization of the seizure onset zone. For 14 of these, this was due to finding a focal cluster of epileptiform discharges, and in one case a regional cluster of discharges. For this group of 15 children, nine had surgery, six of whom had Engel I outcome (77%), two had Engel II outcome (22%), and one had Engel III outcome. The only Engel III outcome was in the child with a regionally dispersed MEG cluster and a GABRG2 gene mutation. The six patients who did not have surgery either declined surgical intervention, or are scheduled for surgery at the time of this publication. In the other 17 cases where MEG contributed to the decision-making process for surgery, the MEG was concordant with other modality data in 11, and provided data that was classified as either supplementary (six cases) or confirmatory (five cases) in the decision-making process, based on review of our group’s Case Conference discussions that took place prior to surgical intervention. Of the “concordant and supplementary” classification cases, five of the six had surgery of which three had Engel I outcome and two had Engel II outcome, and one has surgery scheduled at the time of publication. In four of these, the MEG showed either hemispheric or regional localization, which extended the epileptogenic zone from that suggested by the other data alone, and resulted in decisions toward more extensive surgeries (three hemispherotomies and one POT). In the other two cases, supplementary MEG data also suggested more extensive epileptogenicity, leading to decision to recommend invasive Phase II intracranial monitoring, one of which was declined by family and the other leading to a corpus callosotomy (see section “Clinical Vignette 2”). In the five cases classified as “concordant and confirmatory,” the MEG data contributed by reinforcing data from other modalities. In Case 5, MEG findings confirmed hemispheric epileptogenicity and led to decision for hemispherotomy with an Engel I outcome. In Case 6, MEG findings confirmed bilateral and multifocal epileptogenicity, leading to decision for corpus callosotomy resulting in Engel I outcome. In Case 12, MEG results confirmed regional epileptogenicity resulting in decision for POT, which unfortunately resulted in Engel III outcome. This case then went on to have a hemispherotomy completed at another institution, which has resulted in subsequent seizure-freedom. The other two cases had MEG confirm bilateral multifocal epileptogenicity, leading to decision for VNS placement. Four cases of our cohort had MEG findings that were discordant with the other modality data. In three of these cases, the MEG findings were bilateral and multifocal, and led to decision in one to not recommend surgery (“no-go”). In the other two cases, focal surgeries were performed disregarding the MEG localization, resulting in less successful surgical outcome (Engel III). In the last case with discordant MEG data, MEG revealed a focal cluster concordant with MRI lesion but not EEG findings, which resulted in a decision to recommend Phase II invasive monitoring, subsequently refused by the family.

## Conclusion

In this paper, we report our institutional experience with inpatient MEG in children over an 8-year period, and propose five indications for obtaining inpatient MEG. Clinical MEG is performed predominantly in an outpatient setting in the current United States healthcare environment. However, as seen in our single institution experience, there are clinical circumstances where performing MEG as an inpatient is instrumental in timely decision-making that can result in life-saving care, may increase significantly the likelihood of a successful study, or can contribute to more efficient and superior overall care of the patient. Although availability and circumstances may vary among clinical MEG centers within the United States and outside the United States, it is our hope that these indications can instruct a way of approaching children with intractable epilepsy that takes advantage of the intrinsic value of MEG in optimizing surgical decision-making and formulating more effective treatment plans, in one of the world’s most vulnerable groups of individuals with epilepsy. While further investigation is warranted to evaluate clinically significant long-term outcomes, our clinical experience demonstrates that MEG can be safely and effectively performed in inpatient settings, and can provide essential information regarding the localization of epileptogenic or functional activity that that aids in timely and informed clinical decision-making. In addition, our cohort confirms the usefulness of MEG in surgical decision-making. Our experience demonstrates that in cases with a focal cluster on MEG, more specific localizing data guides SEEG placement or focal resections that result in better surgical outcomes, and in cases with more extensive epileptogenic abnormalities, MEG can clarify the extent of epileptogenicity and thus contribute to decision-making that leads to more effective surgical choices for those children. Even discordant MEG findings can be helpful in decision-making, by leading to “no-go” decisions, Phase II evaluations, or choice of alternative treatments such as VNS or ketogenic diet, or by predicting less effective surgical outcome by indicating a more complex or extensive epileptogenic network.

## Data Availability Statement

The original contributions presented in the study are included in the article/supplementary files, further inquiries can be directed to the corresponding author.

## Ethics Statement

The studies involving human participants were reviewed and approved by University of Texas Health Science Center Institutional Review Board: HSC-MS-17-0092, Surgical evaluation of Pediatric Epilepsy: Analysis of EEG, MEG, FDG-PET, SEEG, and ECoG in localization and seizure outcome after resective surgery. Written informed consent to participate in this study was provided by the participants’ legal guardian/next of kin. Written informed consent was obtained from the minor(s)’ legal guardian/next of kin for the publication of any potentially identifiable images or data included in this article.

## Author Contributions

GV, MF, and MW contributed to conception and design of the study. SG-T, ES, and MW organized the database. MW, GV, and ES wrote the first draft of the manuscript. MW, ES, GV, MF, and JM wrote sections of the manuscript. MF, CL, and ES designed figures and tables. All authors contributed to manuscript revision, read, and approved the submitted version.

## Conflict of Interest

The authors declare that the research was conducted in the absence of any commercial or financial relationships that could be construed as a potential conflict of interest.
